# Design and validation of a mechanically flexible and ultra-lightweight high-density diffuse optical tomography system for functional neuroimaging of newborns

**DOI:** 10.1117/1.NPh.8.1.015011

**Published:** 2021-03-26

**Authors:** Hubin Zhao, Elisabetta M. Frijia, Ernesto Vidal Rosas, Liam Collins-Jones, Greg Smith, Reuben Nixon-Hill, Samuel Powell, Nicholas L. Everdell, Robert J. Cooper

**Affiliations:** a University College London, DOT-HUB, Department of Medical Physics and Biomedical Engineering, Biomedical Optics Research Laboratory, London, United Kingdom; b University of Glasgow, James Watt School of Engineering, Glasgow, United Kingdom; c Gowerlabs Ltd., London, United Kingdom; d Imperial College London, Department of Mathematics, London, United Kingdom; e Nottingham University, Department of Electrical and Electronic Engineering, Nottingham, United Kingdom

**Keywords:** functional near-infrared spectroscopy, diffuse optical tomography, high-density diffuse optical tomography, neonate, dynamic phantom, wearable, flexible

## Abstract

**Significance:** Neonates are a highly vulnerable population. The risk of brain injury is greater during the first days and weeks after birth than at any other time of life. Functional neuroimaging that can be performed longitudinally and at the cot-side has the potential to improve our understanding of the evolution of multiple forms of neurological injury over the perinatal period. However, existing technologies make it very difficult to perform repeated and/or long-duration functional neuroimaging experiments at the cot-side.

**Aim:** We aimed to create a modular, high-density diffuse optical tomography (HD-DOT) technology specifically for neonatal applications that is ultra-lightweight, low profile and provides high mechanical flexibility. We then sought to validate this technology using an anatomically accurate dynamic phantom.

**Approach:** An advanced 10-layer rigid-flexible printed circuit board technology was adopted as the basis for the DOT modules, which allows for a compact module design that also provides the flexibility needed to conform to the curved infant scalp. Two module layouts were implemented: dual-hexagon and triple-hexagon. Using in-built board-to-board connectors, the system can be configured to provide a vast range of possible layouts. Using epoxy resin, thermochromic dyes, and MRI-derived 3D-printed moulds, we constructed an electrically switchable, anatomically accurate dynamic phantom. This phantom was used to quantify the imaging performance of our flexible, modular HD-DOT system.

**Results:** Using one particular module configuration designed to cover the infant sensorimotor system, the device provided 36 source and 48 detector positions, and over 700 viable DOT channels per wavelength, ranging from 10 to ∼45  mm over an area of approximately $60\;{\mathrm{cm}}^{2}$. The total weight of this system is only 70 g. The signal changes from the dynamic phantom, while slow, closely simulated real hemodynamic response functions. Using difference images obtained from the phantom, the measured 3D localization error provided by the system at the depth of the cortex was in the of range 3 to 6 mm, and the lateral image resolution at the depth of the neonatal cortex is estimated to be as good as 10 to 12 mm.

**Conclusions:** The HD-DOT system described is ultra-low weight, low profile, can conform to the infant scalp, and provides excellent imaging performance. It is expected that this device will make functional neuroimaging of the neonatal brain at the cot-side significantly more practical and effective.

## 1 Introduction

Functional near-infrared spectroscopy (fNIRS) is a form of neuroimaging that is non-invasive and non-ionizing. The basic principle on which fNIRS is based is that human tissue is relatively transparent to red and near-infrared (NIR) wavelengths of light (at 650 to 1000 nm), and that in this region oxygenated hemoglobin (HbO) and deoxygenated hemoglobin (HbR) are the dominant absorbers. As a result, fNIRS is able to indirectly monitor functional brain activity by measuring changes in the concentrations of hemoglobins, which is also the basis of the blood-oxygen-level-dependent functional magnetic resonance imaging (fMRI) signal. While fMRI is primarily sensitive to changes in the concentration of HbR, fNIRS has the ability to differentiate between HbO and HbR, providing more complete information on hemodynamics and cerebral oxygenation.

Because fNIRS measurements are obtained by simply applying sources and detectors of NIR light to the scalp, the technique allows functional neuroimaging of the human cortex to be cheap, accessible, easy to use, and available at the bedside or in almost any other environment. Due to these advantages, fNIRS technologies have been successfully applied across multiple neuroscience domains (including studies of social cognition, autism, sleep, multi-person interaction, and language development)^1^^–^^5^ and in the clinic (e.g., in the study of epilepsy, stroke, and traumatic brain injury).^6^^–^^8^

While fNIRS technologies have been widely used in adults, these technologies also have a close and long-term association with studies of the infant and neonatal brain. It has been over 20 years since fNIRS technologies were first applied for monitoring neonatal brain activation.^9^^–^^11^ A key reason for this association is that fNIRS can be used to monitor localized hemodynamic responses in infants and neonates while they are awake and moving with relative freedom.^12^ Unlike other neuroimaging technologies (including fMRI) that require the subject to remain still, fNIRS has demonstrated reasonable tolerance to motion, fundamentally because the head-mounted sources and detectors can (at least partially) move with the subject.^13^ In addition, due to their thinner skulls, smaller craniums, and typically low-density hair, applying fNIRS in infants and neonates can often allow superior light transmission than is seen in adults. Neonates are also a highly vulnerable population. The risk of brain injury is greater during the first days and weeks after birth than at any other time of life.^14^ Common conditions include perinatal stroke and hypoxic ischaemic encephalopathy, both of which can result in seizures and motor disorders such as cerebral palsy.^15^^,^^16^ Behavioural disorders (such as autism and attention deficit hyperactivity disorder)^17^^,^^18^ can also become evident during infancy and early childhood.

There is therefore a need for effective and early investigation of the brain in neonates at risk of neurological injury,^19^^,^^20^ and functional neuroimaging can potentially help to alleviate this need. Applying fMRI to the vulnerable infant is challenging because of the need to move them away from their clinical unit to the MRI suite. At the same time, the quality of functional imaging that can be applied at the cot-side is generally limited. It is therefore very difficult to achieve long-duration or repeated functional neuroimaging of neonates with good spatial resolution with any existing technology, including fNIRS devices.^12^^,^^21^^–^^24^

The most popular multi-channel fNIRS devices are continuous-wave (CW) technologies, which are relatively low cost and straightforward to set up and operate.^12^ However, with the utilization of optical fibre bundles, conventional benchtop-based fNIRS technologies are bulky, cumbersome, and susceptible to decoupling of the fibre and the scalp during motion. Moreover, the number of sources and detectors a system can provide is limited by the physical weight and size of the optical fibre bundles, which in turn limits the effective number of channels that can be applied to the scalp, particularly for neonates.^12^

For these reasons, it has long been a goal in the fNIRS field to push current technologies from bench-top modalities to fibreless and wearable form factors, via miniaturization of the associated optoelectronics. Wearable technologies hold several advantages for experimental applications, including the potential to be applied in naturalistic environments,^25^ in neurotelemetry studies,^26^ to permit unrestricted/untethered recording, and to investigate the cerebral hemodynamic responses to movement itself.^27^ There are now several commercial wearable fNIRS technologies.^28^^–^^32^ However, despite their successes, these technologies are subject to common limitations: a relatively low channel count, low spatial resolution, and little or no depth specificity.

Diffuse optical tomography (DOT) is an extension of fNIRS in which multiple sources and detectors of NIR light are arranged so as to provide overlapping spatial sampling of the target object. The resulting multi-channel data can be used to reconstruct three-dimensional images of changes in the optical properties of that target object. DOT has been applied to study the neonatal brain since the late 1990s,^33^ and has more recently been used to examine the processing of emotional speech,^34^ to characterize resting-state functional connectivity,^35^ and to detect and study electrographic seizures.^24^^,^^36^

In the last decade, it has been shown that using more sources and detectors packed into a “high-density” array can provide improved spatial resolution and specificity relative to fNIRS and traditional DOT approaches.^37^ High-density DOT (HD-DOT) overcomes the limits of low-density fNIRS sampling, but (due to the requirement of an increased channel count) makes the construction of wearable devices even more challenging.

These challenges can now be overcome using the strategy of modular device design:^38^ an HD-DOT module integrates optical sources, detectors, and control electronics within a relatively small footprint, and multiple modules can be connected together to form a dense array that provides both intra- and inter-module measurement channels.^39^ This design architecture allowed our group to produce the first functional images of the human brain using a fiberless, high-density DOT device,^38^ and apply an extension of that same technology to image the brain during overt movement.^39^ The first commercially available modular, high-density DOT device was launched in 2019 (LUMO, Gowerlabs Ltd.).^40^^,^^41^ For neonatal applications, the mechanical and optoelectronic design requirements of a wearable device are even more stringent. Wearable technologies need to be smaller, lighter, and able to conform to the highly curved neonatal scalp.

In recent years, rigid-flexible printed circuit board (PCB) technology has been adopted to construct several wearable fNIRS prototypes.^42^^,^^43^ In these devices, optical components and control electronics (usually located within the rigid areas) are connected via regions of flexible PCB, instead of using cabling. These flexible PCB fNIRS devices exhibit several advantages. They are lightweight and offer a cableless construction, and they can be placed directly over the curve of the subject’s scalp, which potentially permits better conformity, better optical contact, and superior wearability.^44^

Building on this prior work, in this paper, we propose a wearable, modular, rigid-flexible HD-DOT technology that is based on the LUMO platform (Gowerlabs Ltd., UK), but designed specifically for imaging the sensorimotor system of the term and preterm neonate. Our approach yields a system that is significantly smaller and lighter than all existing high-channel-count fNIRS/DOT devices, while also providing a reconfigurable design and the mechanical flexibility necessary to allow each module to conform to the highly curved infant scalp.

To evaluate the conformability and imaging performance of this HD-DOT system, a dynamic optical phantom with accurate neonatal anatomical geometry and precisely localized optical targets is required to simulate human brain activation. Electrically switchable, solid-state optical phantoms have been demonstrated using various approaches.^45^^–^^47^ One such approach is based on the use of thermochromic dyes and was first presented by Hebden et al.^48^ in 2008. Thermochromic dyes are substances that change color with a temperature change above (or below) a specific activation temperature. The dyes typically come in the form of a powder that consists of a mixture of thermochromic pigments and a co-solvent. The pigments are bound in microcapsules (also called leuco-dyes) of 3 to 5  μm in size. These contain the color former and a weak acid. Their reaction with the acid causes a change in the absorption properties, which leads to a coloration/decoloration. The solvent is solid at lower temperature and the colored dye-acid complexes are therefore present. At higher temperatures, the solvent melts, reacts with the acid, and divides the dye-acid complexes causing a loss of, or change in, color.^49^^,^^50^ This reaction is reversible and repeatable several thousand times.^51^ Several industrial thermochromic dyes are available with a range of different activation temperatures and colors that can be mixed with epoxy resins or plastics.

Prior work has shown that thermochromic targets embedded in solid dynamic phantoms can successfully produce controllable changes in optical properties and are an effective approach for simulating functional changes in the human brain. To evaluate the ability of our wearable rigid-flexible modular HD-DOT system to image the infant motor cortex, we designed a new dynamic thermochromic phantom that is based on an infant MRI atlas and overcomes many of the manufacturing and performance challenges of previous phantom designs.

## 2 Methods

### 2.1 Module Design

**Fig. 1:**
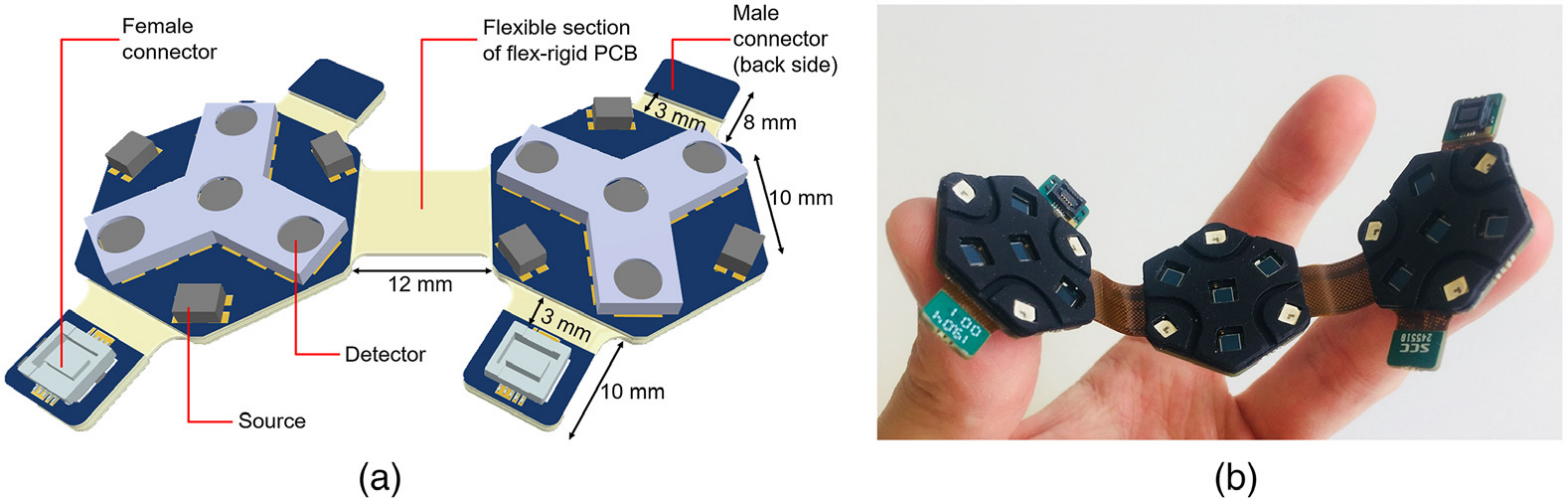
The 10-layer rigid-flexible HD-DOT modules of two different designs: (a) dual-hex module and (b) tri-hex module. (a) A 3D diagram of a dual-hexagon module, each module contains four connector tabs, which include two female connectors and two male connectors (back side). (b) A photograph of the triple-hexagon module, demonstrating its flexibility. The triple-hexagon has the same design as the dual-hexagon module, except for the additional hexagonal board added in the center.

An advanced 10-layer rigid-flexible PCB technology was adopted as the basis for the HD-DOT modules. Two patterns of module were implemented: dual-hexagon (dual-hex) and triple-hexagon (tri-hex) (Fig. 1). Each module consists of a flexible chain of either 2 or 3 regular hexagonal units. Each hexagonal section is based on the Gowerlabs LUMO module and is equipped with 3 dual-wavelength light-emitting diode (LED) sources and 4 silicon photodiode detectors. Each LED package provides two wavelengths of light at 735 and 850 nm, and each silicon photodiode has a photosensitive area of $2.77\times 2.77\;{\mathrm{mm}}^{2}$. Each LED is positioned at a corner of the hexagon to form an equilateral triangle, and three (of four) photodiodes are placed at the remaining three corners. The final photodiode is positioned at the center of the hexagonal unit. This layout generates source–detector separations (SDS) of 10 mm (9 channels) and 20 mm (3 channels) within each hexagonal unit. In each hexagonal unit, the LEDs are controlled by an LED driver and each photodiode is connected to 1 of 4 channels of an analog-to-digital converter (ADC) which records the data in real time. In this system, when illuminated, each LED provides an optical power of approximately 8 mW. A local micro-controller unit provides control for each module. It regulates LED source illumination, fetches optical measurements from the ADC, and writes data into a serial bus.

The dual-hex module yields 48 DOT channels per wavelength over an area of $∼9.4\;{\mathrm{cm}}^{2}$ and the tri-hex module can produce 108 DOT channels per wavelength over an area of $∼14.1\;{\mathrm{cm}}^{2}$. To avoid the need for separate cabling, board-to-board connectors are built into each DOT module and positioned on a dedicated connector tab. In each module, there are four connector tabs, with each housing one male or female connector. A flexible section is present between each connector tab and the adjacent rigid hexagonal unit to provide additional flexibility and articulation of the connector. The length of this flexible section is set as 3 mm, which is sufficient to permit an appropriate bend radius on the basis recommended by IPC-2223 flex-rigid design rules and the minimum radius of curvature expected in the target population (28 week gestational age preterm infant).^52^ The total length of female and male connector tabs are 10 and 8 mm, respectively, which results in a 12-mm cross-module distance when a male and female connectors of adjacent DOT modules are connected. The flexible section between each two hexagonal units in both double-/triple- hexagonal modules is correspondingly set at 12 mm so as to maintain the symmetry of the imaging array when combining multiple modules.

### 2.2 Combining Multiple Modules

**Fig. 2:**
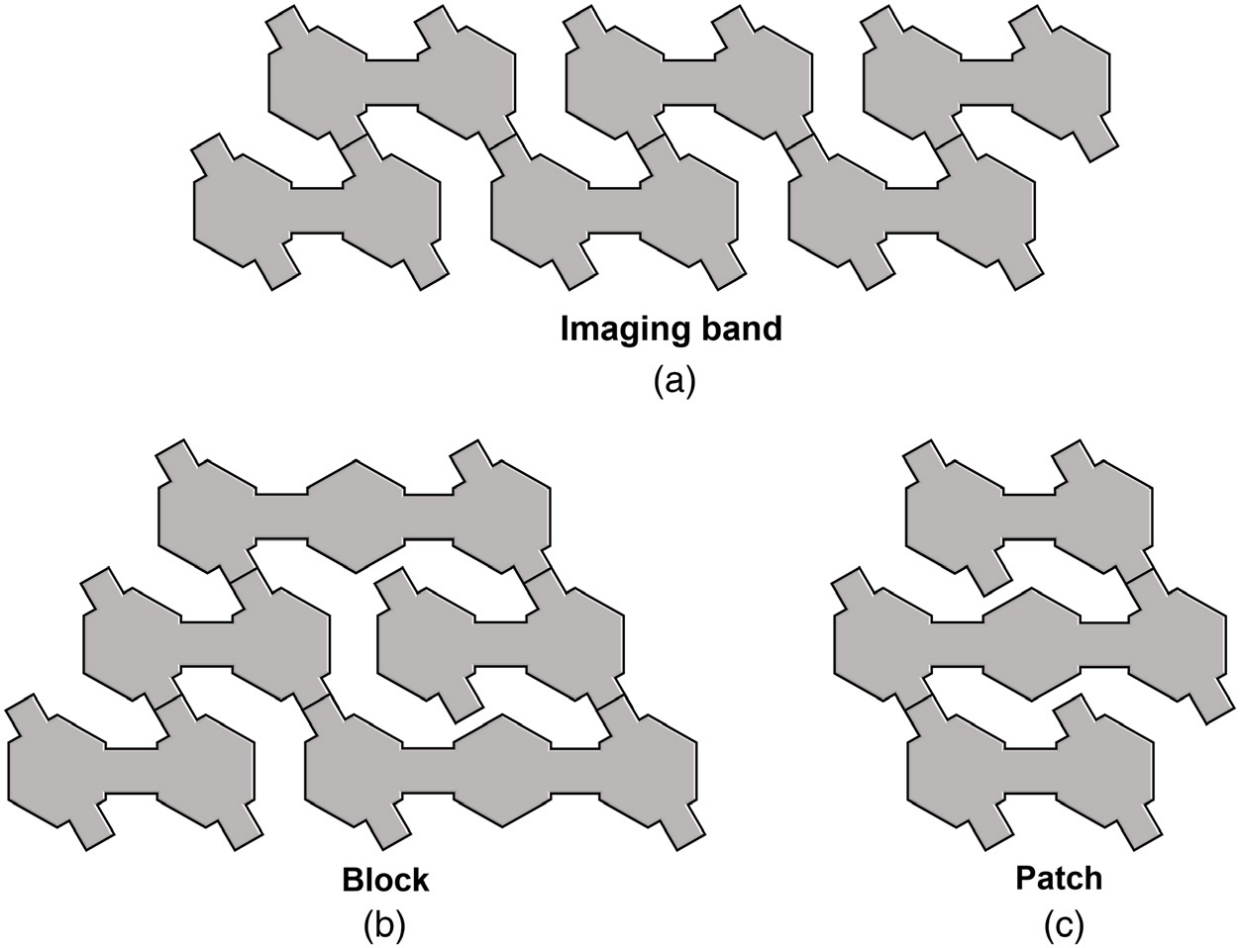
A conceptual diagram of arbitrary module combinations, such as (a) an imaging band, (b) a block, and (c) a patch. Combining different numbers of dual-hex and tri-hex modules, a vast range of possible layouts can be generated via in-built board-to-board connectors.

**Fig. 3:**
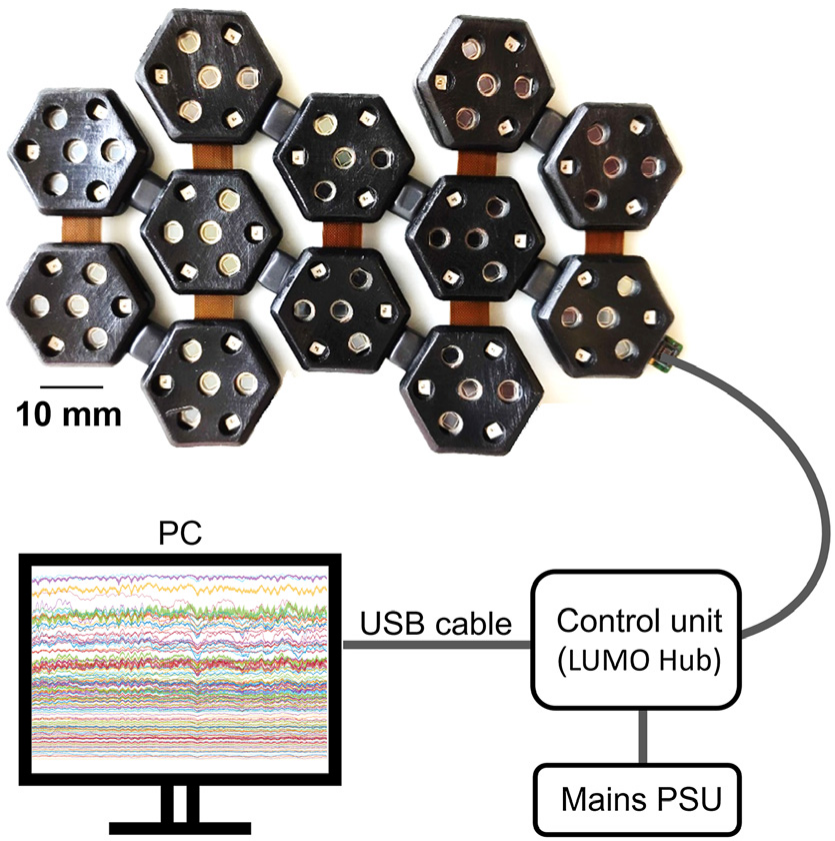
The 12-hex DOT imaging array built using 3 dual-hex and 2 tri-hex modules, with encapsulation. This imaging array adopts a 2-3-2-3-2 layout that can provide appropriate coverage for the motor cortex of neonates. The system configuration of control unit (LUMO hub), PC, and cabling are also depicted (not to scale).

As the dual-hex and tri-hex modules can be connected together via the in-built board-to-board connectors, the system can be configured to provide a vast range of possible layouts. Figure 2 provides some examples of possible configurations. It is noteworthy that if a given connector tab is unused as part of a (permanent) array layout, those tabs can easily be removed. As a first demonstration of this system, we used 3 dual-hex and 2 tri-hex modules with a 2-3-2-3-2 layout configuration as shown in Fig. 3. This module configuration provides coverage of approximately $60\;{\mathrm{cm}}^{2}$, which is sufficient to ensure the sensorimotor cortex of the term-age neonate can be fully covered. This configuration provides 36 source and 48 detector locations and can generate 1728 logical DOT channels per wavelength.

This system is controlled as per the commercial platform developed by Gowerlabs Ltd. The modules are connected to a base station (“hub”) for control and communication (via embedded serial bus) and to supply power using a single lightweight cable. The hub itself is connected to a laptop PC via USB (see Fig. 3). The system operates at a full frame rate of 10 Hz.

### 2.3 Shielding and Encapsulation

**Fig. 4:**
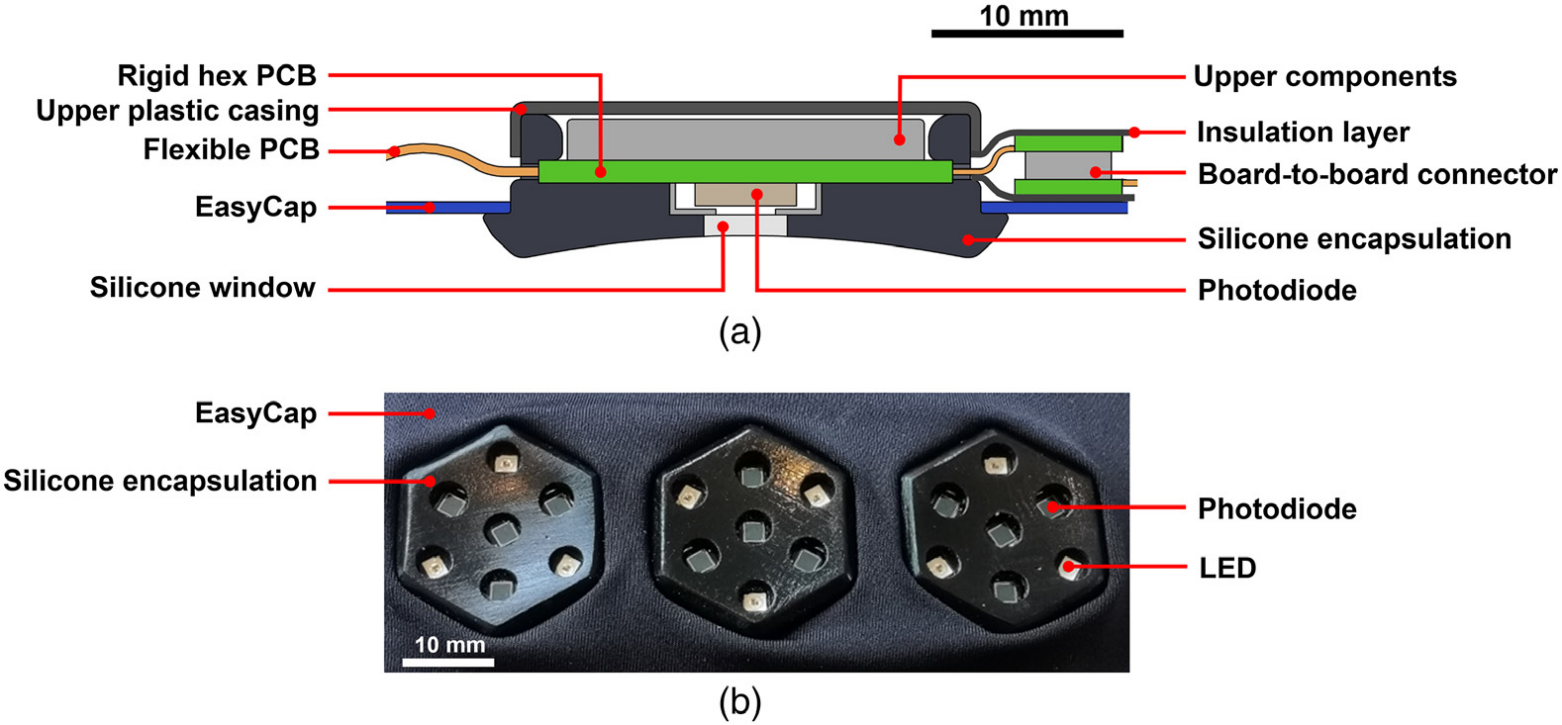
Encapsulation of the individual dual-hexagon and triple-hexagon modules. (a) A cross-sectional diagram of an encapsulated module, and (b) a photograph of a triple-hexagon module with complete encapsulation connected into and EasyCap.

For this neonate-specific HD-DOT system, it was critical to achieve an encapsulation that is lightweight, soft, and comfortable for the infant. It is also necessary that each external component is readily cleanable and/or disposable, to permit application in the clinical environment. Thus, a silicone rubber casing was designed to fit around each hexagonal unit in any orientation (Fig. 4). The black opaque silicone was created using a custom-designed injection moulding tool, with optically clear silicone windows subsequently cast in over the location of each optical component, to provide a permanent bond and a seamless contact surface. The finished individual casing can be stretched over each unit and removed/replaced for disinfection or disposal when necessary. Each casing also contains an external groove all the way around the outside edge to allow the module to be fixed into a stretchable head cap such as an EasyCap (Brain Products, Germany). Each casing is designed with a curved bottom surface so as to provide optimum coupling to the neonatal scalp.

After the encapsulation of the individual modules (either dual-hex or tri-hex), an array can be formed by connecting the modules together with the desired layout via the in-built board-to-board connectors. Plastic insulation was then applied over the connection joints to support further mechanical strength and robustness. After that, the DOT array was mounted into a fabric EasyCap [as per Fig. 4(b)] into which specific holes had been cut to accommodate the silicone encapsulation.

### 2.4 Phantom Design

We began by creating the main body of the phantom. A homogeneous epoxy resin optical phantom was created based on the 39-week infant scalp surface from the 4D neonatal head model developed by Brigadoi et al.^53^ The two-component epoxy resin (Crystal Clear 206, Smooth-On Inc.) was mixed with titanium dioxide particles (Superwhite, Tiranti Ltd., UK) and an NIR dye (Projet 900NP, ICI, UK) in order to obtain a reduced scattering coefficient ${\mu_{s}}^{\prime }=1\;{\mathrm{mm}}^{-1}$ and an absorption coefficient of $\mu_{a}=0.02\;{\mathrm{mm}}^{-1}$ at 800 nm.^54^^,^^55^ After casting, a 3D-printed jig was used to allow precision drilling of two 8-mm-diameter holes into the phantom. The holes were positioned symmetrically about the mid-line of the phantom head and drilled to a depth of 73 mm into the bottom surface of the phantom, such that the bottom of the resulting holes coincided with the surfaces of the left and right pre-central gyri, as determined using the gray-matter (GM) surface mesh of the same 39-week infant head model.

The thermochromic dye used for the dynamic phantom was the “blue-orange” thermochromic powder dye (Changzhou Hali Chemical Co., Ltd., China), with an activation temperature of 43°C. To create solid targets that could be cast in any desired shape, the thermochromic powder was mixed with the epoxy resin (EpoxAcast™ 690), to a concentration of $25\;\mathrm{mg}{\mathrm{cm}}^{-3}$.

**Fig. 5:**
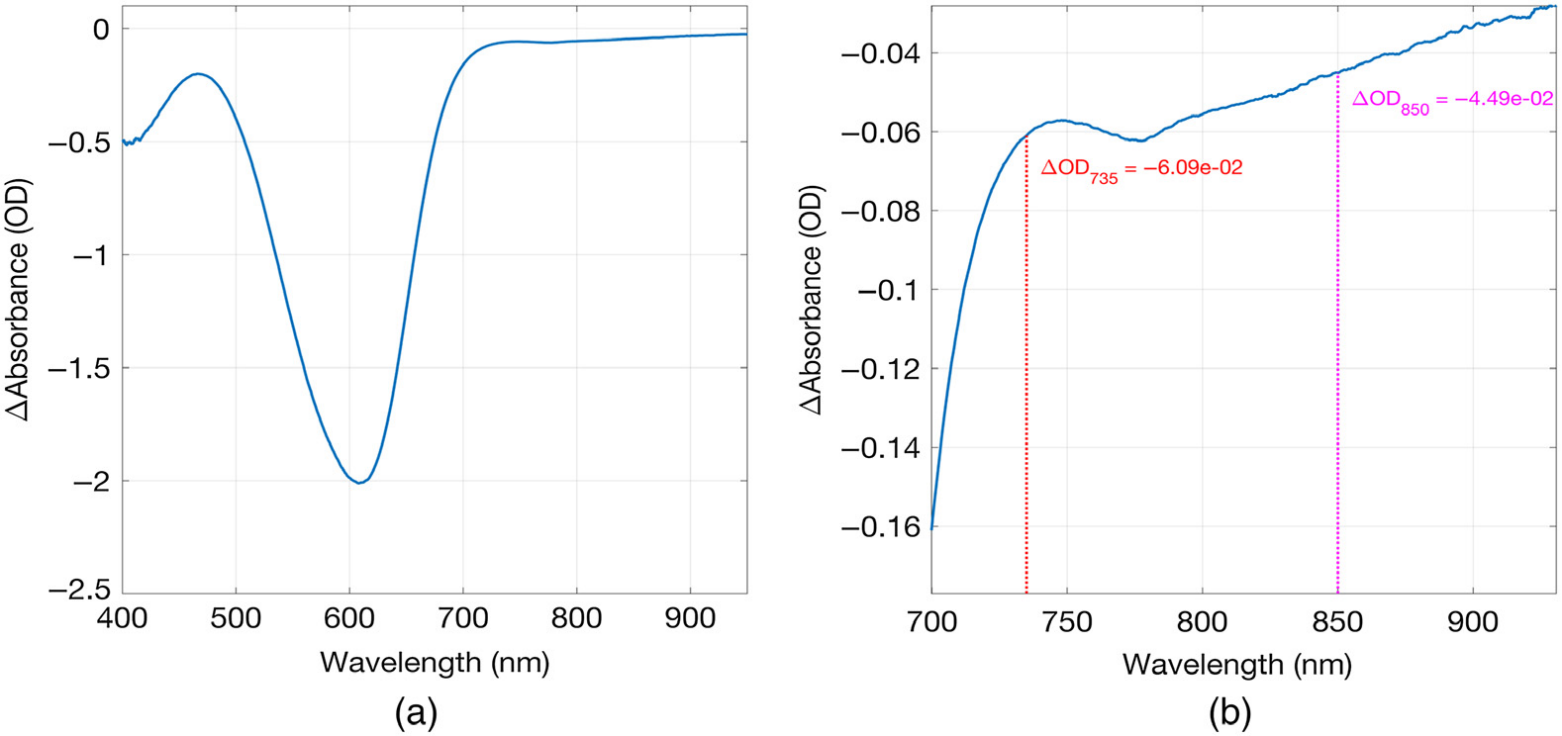
The change in absorbance as a function of wavelength between room temperature (22°C) and 50°C (above the activation temperature of 43°C) for a 3-mm-thick thermochromic sample, with a dye concentration of $25\;\mathrm{mg}{\mathrm{cm}}^{-3}$. (a) The spectral range 400 to 950 nm, and (b) 700 to 950 nm.

Prior to selecting the dye for construction of the phantom, its attenuating properties were examined. Semi-circular disk-shaped samples of 3-mm thickness were cast using a powder concentration of $25\;\mathrm{mg}{\mathrm{cm}}^{-3}$ as already stated. A spectrometer (FLAME, Ocean Optics Inc.) was used to perform transmission measurements through the disk at room temperature and at 50°C to evaluate the changes in absorption spectra before and after heating to above the activation temperature. Light from a tungsten halogen light source (HL-2000, Ocean Optics Inc.) was coupled to the surface of the disk using an optical fibre, with a second fibre returning collected light transmitted through the disk and returning it to the spectrometer. The disk was positioned vertically to allow on a hot plate, which was used to control the temperature of the sample. Another optical fibre carried the emerging light to the spectrometer. Figure 5 shows the change in absorbance (OD) as a function of wavelength for the thermochromic sample. Heating causes a decrease in absorbance that is most prominent at around 600 nm (i.e., in the orange/red region of the visible spectrum, as expected). However, a measurable change is still apparent in the NIR spectrum, and this dye was selected for construction of the dynamic phantom target.

**Fig. 6:**
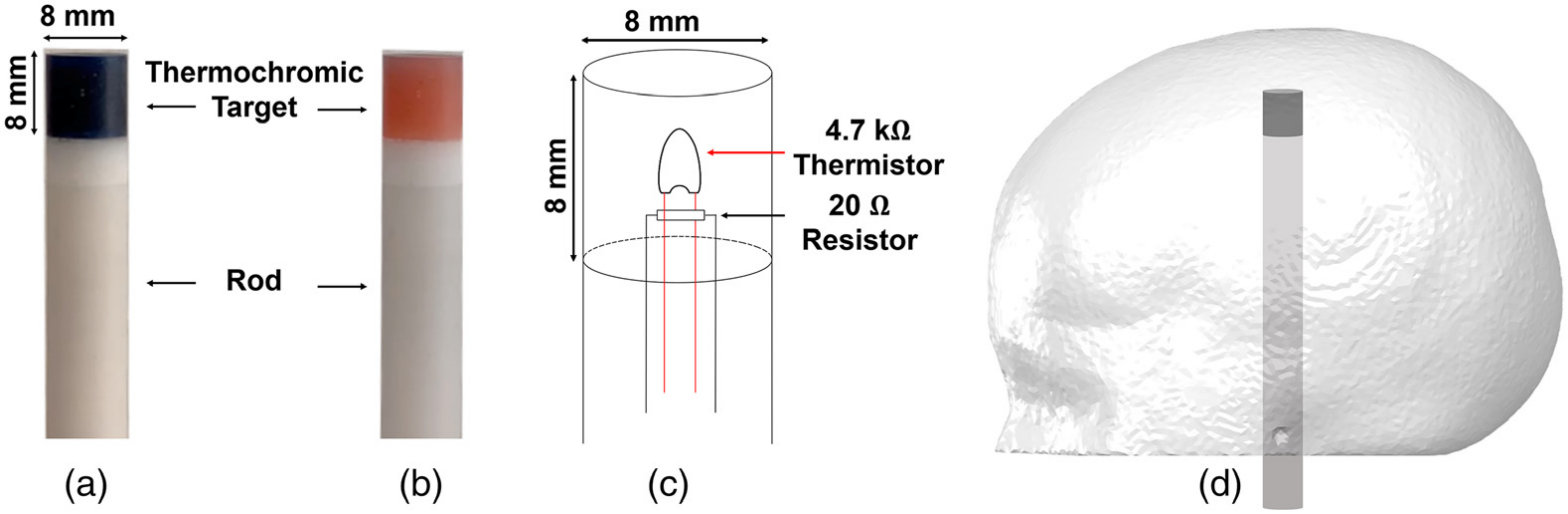
The thermochromic rod at (a) room temperature and (b) above the activation temperature. (c) A diagram of the internal design of the thermochromic rod and (d) an illustration of the phantom and rod.

The targets for the final phantom were created by casting the dye and epoxy resin mixture in a cylindrical polyethylene tube. The samples were left to cure at room temperature for 24 h. After curing and extraction, each sample was machined and refined into an 8-mm-diameter cylinder with a height of 8 mm. A recess of 5-mm diameter was drilled into the end of each target cylinder in order to host a 20-Ω surface mount resistor and a 4.7-kΩ bead thermistor. The resistor dissipates power which yields a rise in temperature, monitored via the thermistor. After the electronic component insertion, the recess was carefully filled with the same dye-resin and left to cure. Two 8-mm-diameter rods of 77 mm in length were also created using a similar method as already mentioned, but using the resin mixture to match the optical properties of the main volume of the head phantom. A small hole was drilled through the center of each rod to act as a cable route. Two thermochromic targets were then glued to the top of each rod using a thin layer of epoxy. This resulted in two cylindrical rods, 8 mm in diameter and 85 mm in length, each with an 8-mm-long thermochromic tip. The phantom and target construction are shown in Fig. 6. An automated control circuit, based on a negative feedback loop that maintains the target temperature just above the transition temperature, was used to control the current through the target circuit, as described previously by Hebden et al.^48^

### 2.5 Phantom Imaging Experiment

This flexible HD-DOT system was used to yield 3D images of the infant dynamic phantom. The array shown in Fig. 3 was placed on the head surface above the two targets, centered over the apex and covering symmetrically the left and right sensorimotor cortices. Cranial landmarks, which were built into the phantom mould, were used as reference points to align the array.

**Fig. 7:**
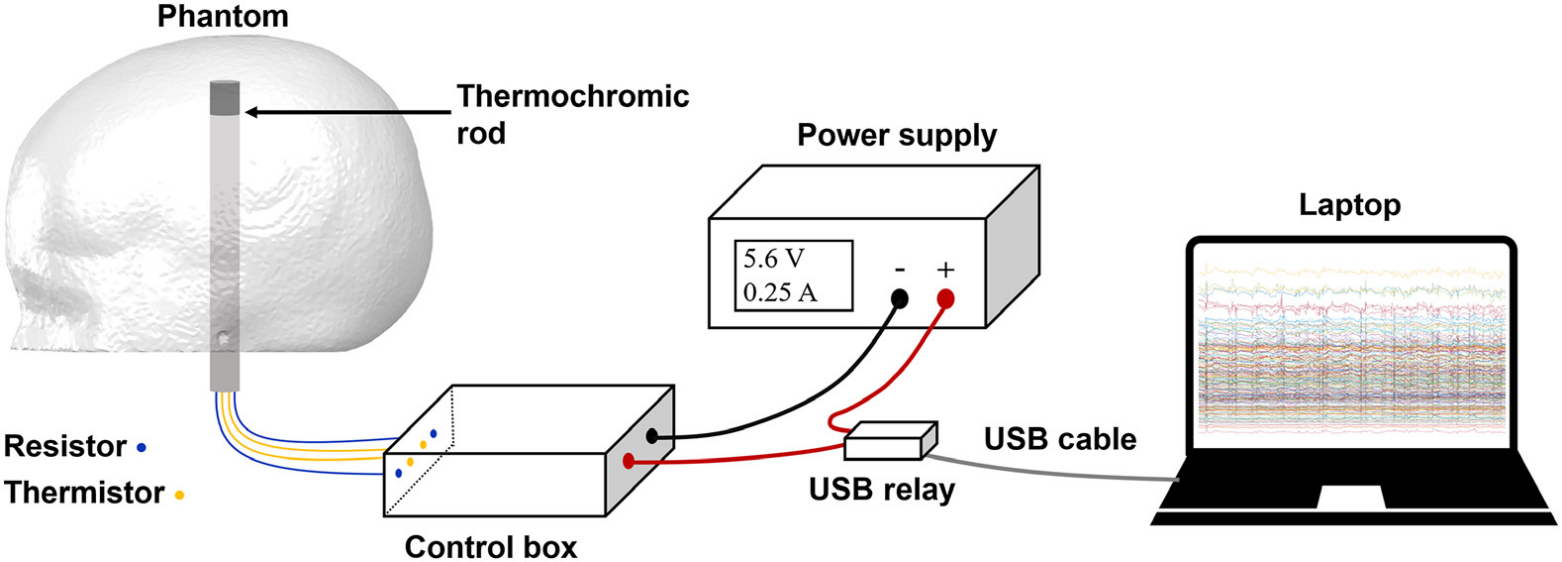
The experimental setup used to control phantom activation experiment. The control box is adopted from Ref. 48 to control the temperature of the thermochromic rod. The power source of the control box is regulated by predefined Matlab script via a USB COM port and the USB relay.

A heating power of 1.4 W was delivered to the rods leading to a change in the optical properties of the target that became evident after approximately 60 s. The target activation was controlled using a USB relay, itself controlled via serial port using Matlab (The MathWorks, Inc.). After a 20-s rest period at the beginning of the recording, the target control circuit was switched on for a period of 180 s, which was followed by 300 s of cooling during which the targets were allowed to return to room temperature. This cycle was repeated 10 times for each target. The experimental setup is shown in Fig. 7.

### 2.6 Photogrammetric Registration

**Fig. 8:**
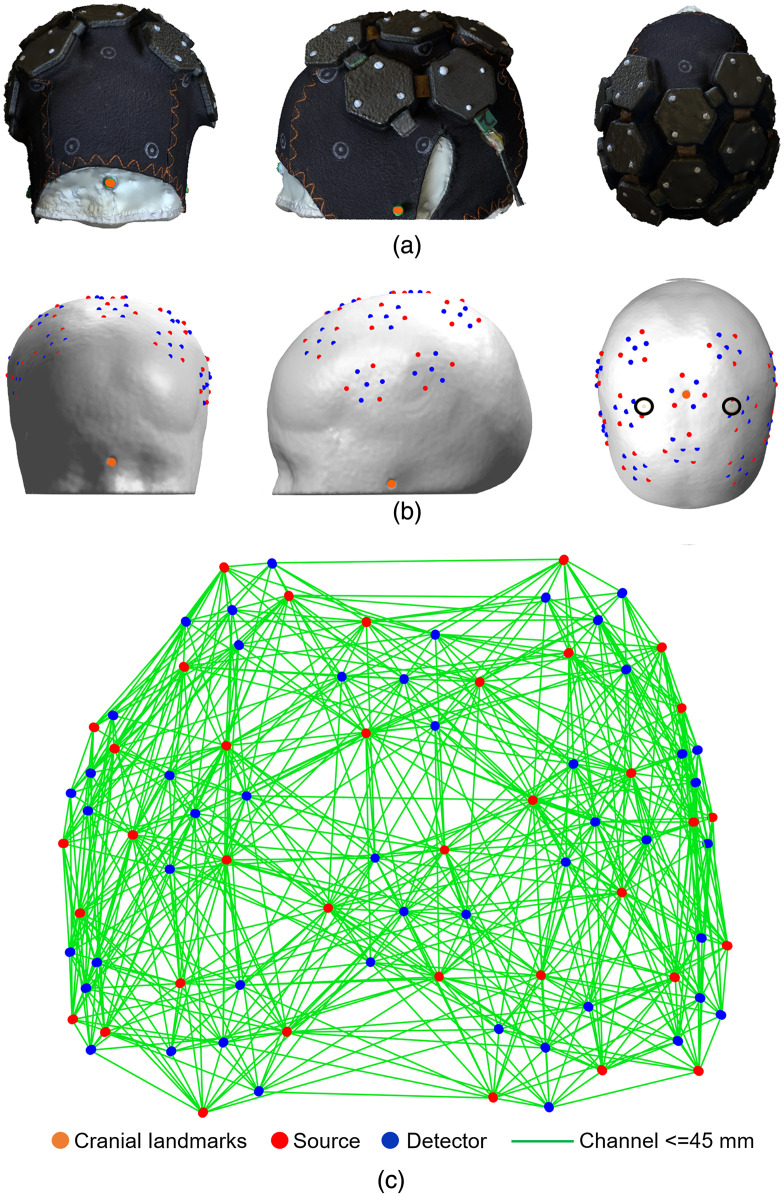
(a) Frontal, lateral, and superior views of the imaging array in place on the dynamic phantom. (b) The corresponding optode positions registered to the anatomical atlas model. The black circles on subpanel (b-3) indicate the rod positions. (c) The 3D channel layout as viewed from above.

A photogrammetric method was used to obtain a three-dimensional model of the phantom head surface with the HD-DOT cap in place. A video was recorded while panning around the phantom at three different heights using the camera of an X-series iPhone (Apple, Inc., California). The movie file was imported directly into Metashape^56^ to create a 3D model, which was exported in the .PLY format. To identify the exact location of sources and detectors, each module was tagged with markers at the points directly above the three sources. Images of the resulting mesh model of the array and phantom are shown in Fig. 8. The cranial landmarks and the source marker locations were determined from the point cloud using the software CloudCompare (Open Source Project^57^). As the dimensions of the module encapsulations are known precisely, the locations of every optode could be determined without further approximation. The resulting 3D array positions are also shown in Fig. 8.

### 2.7 Data Pre-Processing

The data processing consisted of the following steps. First, channels were rejected if their coefficient of variation exceeded 8%.^58^ The data were then converted to absorbance and low-pass filtered. Given the phantom activation cycle lasted 500 s, the low-pass filter was set to 0.01 Hz and implemented using a finite impulse response digital filter.

A detrended block-average was performed to obtain relative changes in absorbance for 500-s-long epochs starting from 20 s before the beginning of each heating cycle. The average of all the 20-s periods of data centered on the time at which the maximum change was observed (200-s post-onset) was used to reconstruct DOT images relative to the baseline period. Three-dimensional images showing the changes in the optical absorption coefficient due to the activation of the targets were thus obtained for both wavelengths.

Channel-wise changes in “hemoglobin concentration” were also obtained by treating the dynamic changes recovered from the phantom as if they were human physiology by applying the modified Beer–Lambert law,^59^ with a DPF=6 for both wavelengths^60^ to the block-averaged absorbance data. This was performed to examine how closely the phantom mimicked the types of signal morphology typically observed in a functional neuroimaging experiment.

### 2.8 Image Reconstruction

Image reconstruction was undertaken in a two-layer mesh model of the infant head. This model included a volume mesh with extracerebral and brain tissue layers, and separate scalp and GM surface meshes, which were derived from the Brigadoi atlas^53^ for the 39-week post-menstral age point. With the exception of the target, the phantom optical properties are homogenous; a two-layer model was used simply to enable the extraction of images at the level of the GM. Both layers of mesh model were modeled with identical optical properties. The mould in which the phantom head was cast was based on the same dataset used to create our meshes, so they are expected to provide a near-perfect model. Once the mesh was registered to the phantom’s cranial landmarks and optode positions derived from the photogrammetry model, TOAST++^61^ was used to compute a forward model for each wavelength and a zeroth-order Tikhonov regularized reconstruction was performed with a regularization hyperparameter of $1\times 10^{-3}$. Images were reconstructed in the full mesh volume and for visualization purposes, image values at the level of the GM surface were also extracted.

## 3 Results

### 3.1 System Performance and Data Quality

The use of 10-layer rigid-flexible PCB technology allows this HD-DOT system to exhibit an ultra-compact circuit layout and a miniaturized profile, and consequently a very lightweight construction. The side of each rigid hexagonal unit is 10-mm long, and the area of modules are $9.4\;{\mathrm{cm}}^{2}$ for the dual-hex module and $14.1\;{\mathrm{cm}}^{2}$ for the tri-hex module. The weight of the dual-hex and tri-hex modules are 8 and 12 g respectively, and the total weight of the imaging array (combing with 3 dual-hex and 2 tri-hex modules and encapsulations) is 70 g. The array configuration shown in Fig. 3 provides coverage of approximately $60\;{\mathrm{cm}}^{2}$, which is sufficient to cover the entire sensorimotor cortex of the term-age neonate, as shown in Fig. 8.

**Fig. 9:**
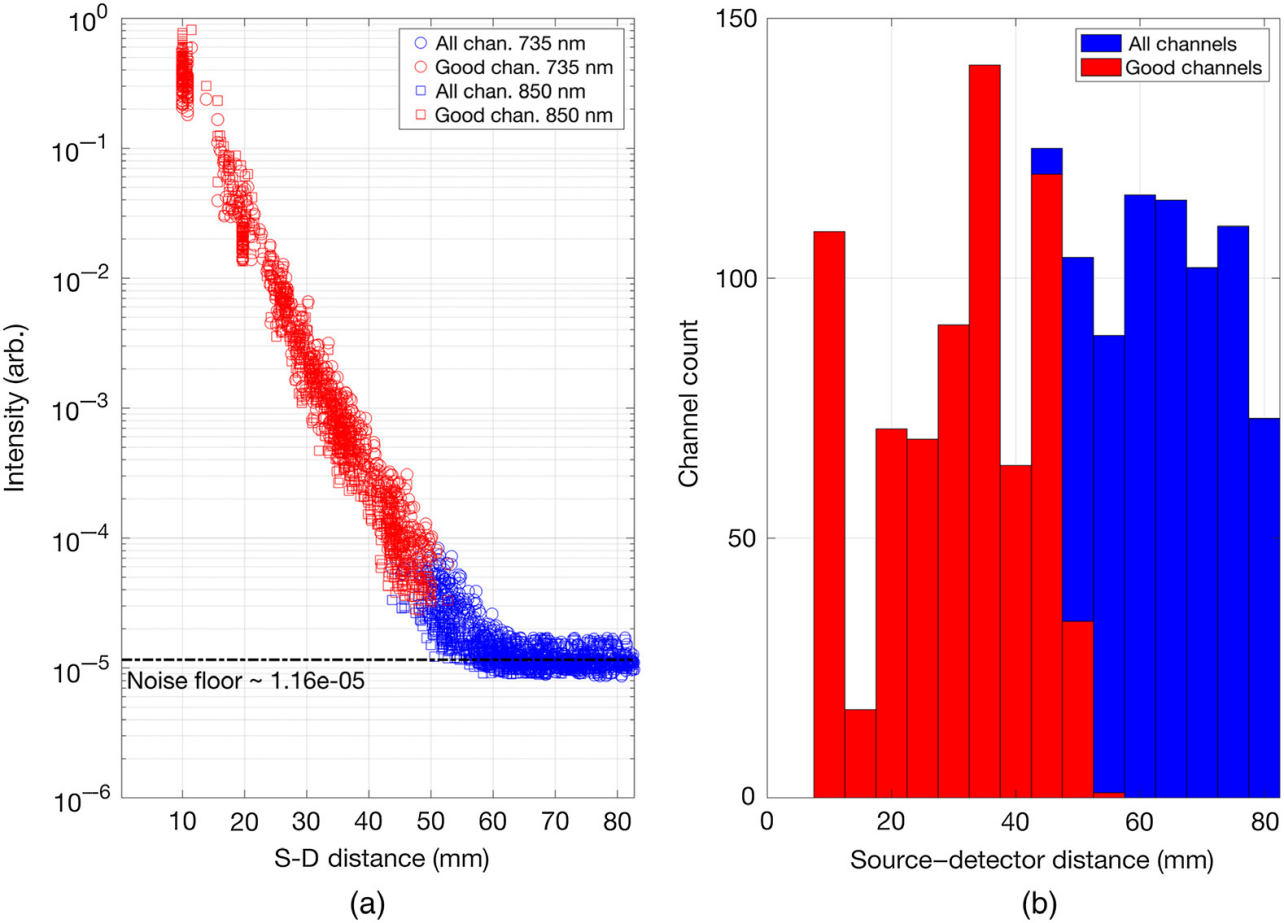
(a) The channel optical intensity measurements as a function of their SDS. Channels that passed out pre-processing thresholds are depicted in red. (b) Histograms of all possible channels (blue) and accepted channels (red) generated on the dynamic phantom as a function of the SDS.

Figure 9(a) shows the optical intensity measurements as a function of SDS. The noise floor is measured to be $1.16\times 10^{-5}$ (arbitrary intensity units), which given the upper measurement limit is 2.5 (arbitrary intensity units) implies a measured dynamic range of 106.6 dB. Figure 9(b) shows the histogram of all possible channels and the accepted channels (good channels) generated on the dynamic phantom as a function of SDS. There are 1728 DOT channels in total per wavelength with 717 good channels per wavelength.

### 3.2 Channel-wise Dynamic Responses

**Fig. 10:**
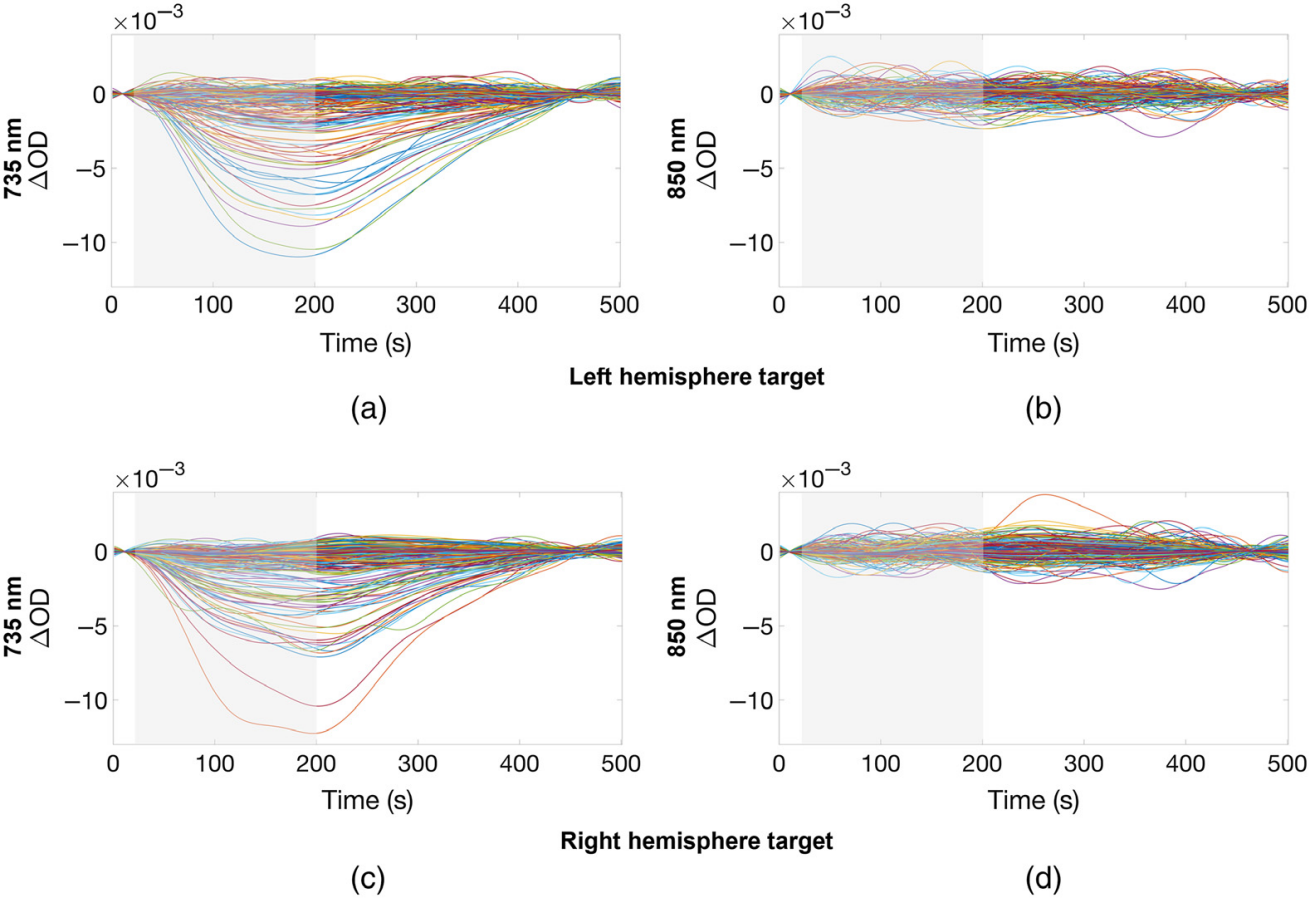
The change in absorbance (optical density) as a result of the activation of the left hemisphere target for the (a) 735-nm and (b) 850-nm wavelengths, and the right hemisphere target for the (c) 735-nm and (d) 850-nm wavelengths, respectively. The heating period is indicated by the gray-shaded area.

The response to the phantom target activation in the case of the left and right activations is shown in Fig. 10 for the two wavelengths used by the system. The channels that exhibited a response show clear changes in optical density with the latency to peak of approximately 160 to 180 s from the beginning of the activation (heating phase). The changes in absorbance are clear in several channels at 735 nm. Consistent changes are not apparent at 850 nm in this channel-wise view.

**Fig. 11:**
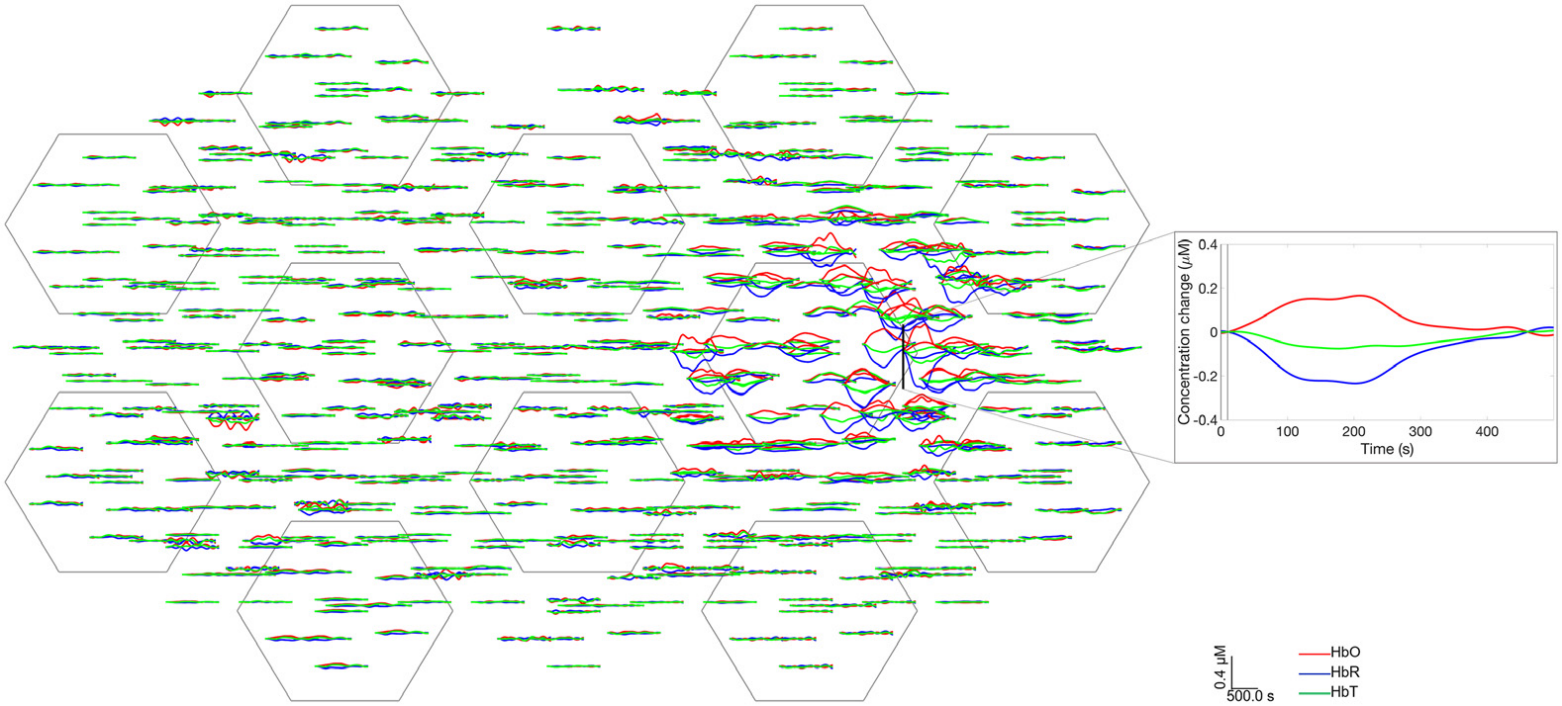
Right hemisphere activation responses in a representative 2D array. It is noteworthy that a high number of channels which exhibit clear responses in the location correspondent to the right rod position show increase in HbO, decrease in HbR as per functional HRF in human brain.

Changes in hemoglobin concentration were considered to examine how well the thermochromic targets can mimic functional hemodynamic responses. Figure 11 shows the simulated hemodynamic response functions (HRFs) displayed on a representative 2D channel layout. The responses exhibit a similar morphology to real HRFs, with the simulated HbO increasing and HbR decreasing. Unlike real HRFs, the simulated HbR response shows a larger magnitude change than HbO. Figure 11 also demonstrates clear, localized changes were obtained for the right-hemisphere activations over the expected locations. (Note: to provide a better fit to the paper format and a more clear display, we decided to cut out the left-hemisphere activations from Fig. 11 and show only one hemisphere.)

### 3.3 Volume and Gray Matter Image Results

**Fig. 12:**
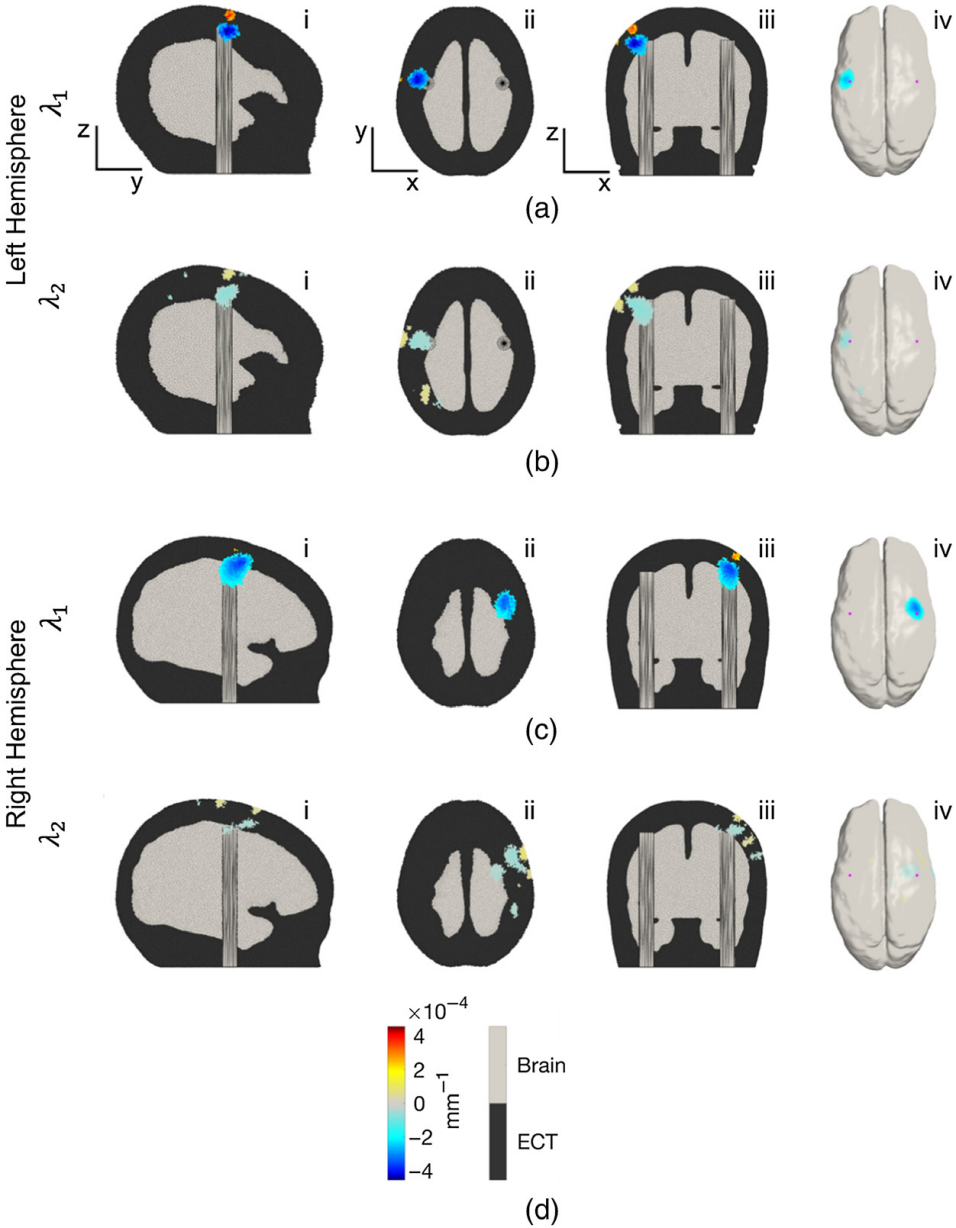
Volumetric and GM -surface images of the change in absorption coefficient at 735 nm ($\lambda_{1}$) and 850 nm ($\lambda_{2}$) displayed for (a), (b) the left and (c), (d) the right hemisphere activations. Images are an average of a time window of between 190- and 210-s post-activation onset, and reconstructed relative to the mean of the 20-s pre-activation. Each image is thresholded at 50% of the maximum absolute signal deviation found in the volume. The sub-panels in each row show the volume images in (i) sagittal section, (ii) transverse section, and (iii) coronal section, and (iv) an extraction of the GM surface.

Three-dimensional images of the change in absorption coefficient at the two wavelengths used by the system were reconstructed in the two-layer neonatal head mesh. Figure 12 shows a sagittal, axial, and coronal view, and the GM surface extraction of the phantom targets. The slices selected for the orthogonal views correspond to the coordinates of the node exhibiting the largest decrease in absorption coefficient at either wavelength in each activation. As is evident in Fig. 12, the peak changes occur with good spatial correspondence to the expected location of the thermochromic targets, over the pre-central gyrus of each hemisphere. The changes obtained at 735 nm are noticeably larger than those at 850 nm, which is consistent with the absorbance changes shown in Fig. 10. To quantify the localization error associated with these images, we computed the offset of the true center of the absorption change in the rod from the center of mass of the spatial distribution of absorption change. The center of mass was computed for each image of the change in absorption coefficient at each wavelength in each hemisphere. First, the image values where thresholded at 50% of the maximum absolute value found across the whole volume. The remaining image values at each node were then multiplied by the Voronoi volume associated with that node and a weighted sum, using the Voronoi volume-corrected absorption change values as a weight, was computed to find the coordinate of the center of mass. The Euclidean separation between the center of mass and the center of the superior face of the rod was computed, as was the Euclidean separation between the center of mass and the center of the rod target volume. The results of this analysis are shown in Table 1. Using the images shown in Fig. 12, the FWHM of the perturbation in x (left-right), y (anterior-posterior), and z (inferior-superior) were also determined at 735 nm. The resulting values were 12.59, 12.04, and 10.52 mm, respectively, for the left hemisphere target, and 12.14, 16.38, and 17.68 mm for the right hemisphere target. The true dimensions of the thermochromic target in these dimensions were 8, 8, and 8 mm, respectively. Because of the multiple distinct regions of change in absorption coefficient in the 850-nm images, we did not attempt to determine FWHM values. In addition, given the relatively low signal-to noise ratio (SNR), the center of mass values at 850 nm are likely unreliable; as such, we consider the 3D Euclidean localization error to range from 3 to 6 mm based on the values obtained from the wavelength at 735 nm.

**Table 1:** Euclidean offset distances between the upper surface of the rod and the center-of-mass of the volumetric image and the middle of the rod and the center-of-mass of the volumetric images for the left and right hemisphere targets at 735 nm, which showed the best contrast.

Wavelength/target	$\lambda_{1}$		$\lambda_{2}$	
	Left hemisphere (mm)	Right hemisphere (mm)	Left hemisphere (mm)	Right hemisphere (mm)
Offset from center of superior surface of target	5.67	3.39	7.81	4.32
Offset from center of target volume	5.83	3.68	6.91	2.59

## 4 Discussion and Conclusions

In this paper, we have described a wearable, HD-DOT system designed specifically for neonatal applications that combines the merits of both modular^38^^,^^39^ and flexible-PCB systems.^42^^,^^43^ The utilization of the 10-layer PCB process results in a highly compact DOT module. All the control electronics and optoelectronic components are located and embedded into the 10-layer rigid island of each DOT module to enable an ultra-low profile and lightweight form factor, while the 4-layer flexible sections are utilized to provide robust connections, stable data transmission, and to enable the system to conform to the neonatal scalp. The 10-layer process also provides additional shielding, which likely further improves the electromagnetic noise susceptibility of the device. The system presented here provides a small footprint, a low profile, a large channel count, and a high channel density, and is generally superior to prior flexible-PCB fNIRS devices.^42^^,^^43^

The mechanical design of the dual-hex and tri-hex modules, each with in-built board-to-board connectors, allows the system to be reconfigurable into a vast range of possible imaging array layouts. Using a 2-3-2-3-2 module layout as shown in Fig. 3, an imaging system was constructed which contains 36 source and 48 detector locations. These values exceed those of most previous wearable devices,^29^^–^^32^^,^^42^^,^^43^ and this large number of source and detector locations provide a larger channel count than the HD-DOT devices used in previous neonatal studies.^62^^,^^63^

The phantom we demonstrate here provides switchable, repeatable, and localized changes in absorption coefficient in the NIR range, and by exploiting MRI data and 3D printing, the construction process ensures anatomically realistic dimensions. The use of removable rod targets makes the manufacturing process significantly more straightforward than previous designs that permanently embedded the targets^48^ and allows for the targets to be replaced as required. This is important because in the event that the embedded circuit fails, or the dye becomes saturated (which can occur if overheated), it is not necessary to rebuild the entire phantom. Unfortunately, the range of available thermochromic dyes, including the dye we selected, produce a relatively small change in absorption coefficient in the NIR range. This is primarily due to the fact that thermochromic dyes are designed to manifest color changes in the visible range, rather than the NIR. As can be seen in Fig. 10, a change in optical properties is barely apparent at 850 nm. The change at 735 nm is small, but with a peak of $∼1\times 10^{-2}$ OD. This is also consistent with Fig. 5, which depicts the absorbance change spectrum provided by the selected thermochromic dye, and shows that the change is expected to be significantly larger at 735 nm than at 850 nm, an effect that is likely to be significantly exacerbated by the relatively broad emission spectrum of LEDs, which were used to provide the NIR light sources in this HD-DOT system. The bandwidth of the LED source is likely the reason that the difference in absorbance changes at 735 and 850 nm are much more apparent in Fig. 10 (when measured with the LEDs of our HD-DOT system), than when measured with the spectrometer (Fig. 5).

Because the change in absorbance is relatively small, multiple repetitions of the heating/cooling cycle were undertaken to ensure adequate SNR. This is time-consuming because the response is ∼10× slower than real functional hemodynamics, but does mimic the approach taken to imaging human brain function. While the phantom we present here does provide switchable optical properties, it of course cannot mimic the significant and widespread physiological noise sources present in a real fNIRS and DOT experiments. The simple single-target nature of this phantom allows for controlled imaging performance testing, but can never fully mimic an *in-vivo* application.

As shown in Fig. 11, one interesting feature of our choice of thermochromic dye is that if the activation data from our dynamic phantom are treated as if it were *in-vivo* functional data, the resulting “simulated” changes in HbO and HbR present a similar morphology to those of functional hyperaemia in the human brain: an increase in HbO and a concomitant decrease in HbR. While of course this linear transformation is physically meaningless in a phantom that contains no hemoglobin, it is potentially useful because it allows end-to-end testing of fNIRS and DOT processing pipelines and software that are designed to yield hemodynamic responses.

One significant limitation of the phantom presented here is that the optical properties of the phantom require more precise characterization. The optical properties of the main body of the phantom are taken from an established recipe,^54^^,^^55^ rather than an experimental measurement of this specific build. The optical properties of the thermochromic phantom targets (both at room temperature and above the activation temperature) also require more precise calibration. It remains unclear to what extent the optical properties of the thermochromic targets differ from those of the main body of the phantom in the NIR range at room temperature, and it is unclear whether activation of the targets generate a change in optical scattering as well as optical absorption. The application of a time-domain NIRS system to measure ${\mu_{s}}^{\prime }$, $\mu_{a}$ and changes in ${\mu_{s}}^{\prime }$ and $\mu_{a}$ during the heating process would enable a more complete characterization of the phantom. While this limitation is less relevant for the application of CW-DOT devices (particularly if the goal is to quantify the upper bounds of image quality), we intend to pursue this characterization when access to time-domain NIRS devices become possible again in 2021.

The data of Fig. 9 demonstrate that the flexible HD-DOT system we present here provides a measured dynamic range of 106.6 dB, which permits acquisition of SDS ranging from 10 to ∼45  mm. This compares favorably to the HD-DOT devices used in previous neonatal studies.^62^^,^^63^ The system generates 1728 logical DOT channels per wavelength and produced 717 viable DOT channels per wavelength when applied to our novel dynamic phantom. This high-density imaging array yielded depth-resolved three-dimensional images with good spatial precision (a 3D Euclidean localization error of between 3 and 6 mm at 735 nm) and (based on the FWHM of the image perturbations at 735 nm) a lateral image resolution of approximately 10 to 12 mm at the depth of the newborn cortex. This also compares well with estimates of spatial resolution obtained in previous HD-DOT simulation studies^64^ and *in-vivo* recording.^37^ It is noteworthy that the reconstructed change in absorption coefficient shown for 735 nm in Fig. 12 appears to be more shallow that the actual target. Furthermore, Fig. 12(a) shows a clear superficial increase in absorption coefficient. We believe that both of these features are artifactual and are related to known limitations of the linear image reconstruction procedure we employed. We believe these features could likely be improved through the application of nonlinear reconstruction schemes, such as the iterative conjugate-gradient method^65^ or quasi-Newton methods.^66^ Reconstruction artifacts are more noticeable for the perturbation at the right-hemisphere, particularly for the wavelength at 850 nm, due to the low SNR of the absorption change [Figs. 10(b) and 10(d)], which could potentially be remedied by increasing the number of repetitions of the target activation cycle.

Even with these caveats, the level of spatial localization and resolution demonstrated should be sufficient to resolve functional responses to the stimulation of each limb in the newborn infant.^67^ In addition, given that the total weight of a 12-hex implementation of this neonatal-specific system is only ∼70  g, this system should allow for significantly improved subject comfort, extended recording periods, and potentially improved motion tolerance relative to existing technologies.
